# Transforming molecular neuropathology for adult brain tumor patients in the UK: Insights on implementation, adoption, and patient access (2021-2024)

**DOI:** 10.1093/nop/npaf099

**Published:** 2025-09-25

**Authors:** Kathreena M Kurian, Andrew P Wright, Fereshteh Sari, Mayen Briggs, Camille Goetz, Ros Quinlivan, Katie Bushby, Richard Gilbertson, Nicky Huskens, Tessa Jowell Centre of Excellence for Adults Committee, Caroline Barry, Mayen Briggs, Carly Butler, Shona Floyd, C Oliver Hanemann, Catharine James, Sarah Jefferies, Michael D Jenkinson, Joanne Lewis, Sara Melhuish, Jess Mills, Nicola Peat, Debi Oliver, Charlotte Robinson, Jillian Sokratous, Adam Waldman

**Affiliations:** Brain Tumour Research Centre, Bristol Medical School, University of Bristol, Bristol, UK; Tessa Jowell Brain Cancer Mission, London, UK; Tessa Jowell Brain Cancer Mission, London, UK; Cambridge University Hospitals NHS Foundation Trust, Cambridge, UK; Tessa Jowell Brain Cancer Mission, London, UK; University College London Hospitals NHS Foundation Trust, London, UK; Tessa Jowell Brain Cancer Mission, London, UK; Cancer Research UK Cambridge Centre, Cambridge, UK; Tessa Jowell Brain Cancer Mission, London, UK

**Keywords:** brain tumors, genomics, molecular neuropathology, UK

## Abstract

BackgroundMolecular neuropathology is evolving rapidly, driven by novel genomic technologies, national policies, and the urgent need for precision brain tumor care. Inequity in access to genomic testing highlighted in previous reports warrants further investigation.

**Methods:**

We analyzed anonymized data from 21 United Kingdom (UK) neuro-oncology centers (covering an estimated 84% of the population) collected in 2021 and 2024 via the Tessa Jowell Centre of Excellence for Adults program. We assessed trends in genomic testing (including methylation arrays, gene panels, whole genome sequencing [WGS]), tumor snap freezing, turnaround times (TATs), and auditing.

**Results:**

From 2021 to 2024, total genomic tests submitted across the 21 centers increased 128% from 2946 to 6730, while glioma cases increased 18% (from 3159 to 3722). Methylation arrays, gene panels, and WGS samples submitted rose by 342%, 174%, and 291%, respectively. Centers requesting WGS increased from 4/21 (19%) to 15/21 (71%), with 90% centers (19/21) reporting snap freezing of brain tumor samples in 2024 (mean 173, range 0-650 samples frozen per center). Mean molecular diagnosis TATs rose from 16 to 21 days; centers meeting a 14-day TAT dropped from 48% (10/21) to 30% (6/20). Auditing of TATs increased from 6/21 (29%) to 18/21 (86%).

**Conclusions:**

UK molecular neuropathology testing grew substantially 2021-2024. However, variations in testing and snap freezing, and bottlenecks in diagnostic TATs, emphasize the need for: (1) targeted training and investment in rapid technologies to ensure sustainable service delivery; (2) focus on remaining inequities and (3) continued engagement with national benchmarking initiatives.

Key points

- Brain tumor molecular diagnostics has grown substantially in the UK 2021-2024
- Inequity between regions in testing, sampling, and turnaround times persist
- Several actionable recommendations would improve equity of molecular testing

Importance of the Study

Molecular neuropathology is advancing rapidly worldwide, yet the extent to which this progress benefits brain tumor patients remain uncertain. This study presents the first United Kingdom (UK)-wide evaluation of molecular diagnostic adoption following the 2021 WHO classification, which established molecular profiling as a global clinical standard in brain tumor care. It provides a detailed analysis of implementation between 2021 and 2024, including gene panels, methylation arrays, whole genome sequencing, tumor snap freezing, and diagnostic turnaround times. Findings reveal significant regional disparities in access, with major gaps in standardization and systemic barriers to progress. While focused on the UK, the insights and challenges identified are relevant to many healthcare systems and offer a blueprint for improving molecular diagnostics internationally. With new brain tumor trials increasingly requiring whole genome sequencing for enrolment, this research highlights the urgent global need for equitable access to deliver precision medicine to all brain tumor patients.

Molecular neuropathology in the United Kingdom (UK) has undergone a transformation over the past two decades, driven by advances in genomic technologies, new biomarker discovery, policy-driven initiatives and the increasing need for precision medicine in brain tumor treatment. The integration of methylation arrays, next-generation sequencing (NGS) panels, and whole genome sequencing (WGS) have redefined brain tumor diagnostics, prognostics, and prediction,^1^^,^^2^ increasingly shaping personalized treatment strategies across brain tumors, neurodegenerative diseases, and rare genetic conditions.^3^ The 2021 WHO classification of central nervous system (CNS) tumors cemented molecular diagnostics as a new clinical standard, marking a pivotal shift in neuro-oncology away from histology alone,^1^ while the identification of isocitrate dehydrogenase (IDH) mutations, 1p/19q co-deletion, and DNA methylation profiling has significantly improved tumor classification and patient stratification.^2^^,^^4^ Beyond conventional short-read NGS, methylation arrays, and WGS, emerging long-read sequencing via nanopore technology offers real-time genomic and epigenomic sequencing with higher throughput and improved cost efficiency.^5^

Alongside technological advancements, policy-driven initiatives have played a critical role in embedding molecular neuropathology into routine clinical practice in the UK. The existence of a national, free at the point of use National Health Service (NHS) has allowed rapid implementation of genomics policy for every patient in the UK; for example, the NHS Genomic Medicine Service and the 100 000 Genomes Project (2012-2018) have both significantly contributed to integration of genomics into everyday clinical practice.^6^ Further, the introduction of the Genomic Laboratory Hub (GLH) system, where genomic testing has been centralized in regional hubs in England, has revolutionized access to key neuropathological testing in brain tumors.

Despite these developments, evidence suggests that there is variation in testing across UK nations and regions. The Tessa Jowell Brain Cancer Mission (TJBCM), a non-profit convening body for the UK brain tumor community, published the Closing the Gap report in 2024,^7^ which highlighted inequalities in genomic testing access across neuro-oncology centers, identifying systemic barriers and possible drivers of this inequity.^7^

One key barrier to wider access to molecular and genomic testing is the ability to snap freeze tissue.^8^ In neuro-oncology, flash-frozen tissue stored at −80 °C is often essential for molecular and genetic analyses, as it preserves DNA and RNA integrity more effectively than formalin-fixed paraffin-embedded (FFPE) samples, which is suboptimal for certain molecular applications due to chemical-induced degradation, fragmentation, and cross-linking of nucleic acids.^8^ As well as enhancing diagnostic accuracy, access to frozen tissue is particularly important to support the urgent need for novel brain tumor therapies and clinical trials (such as vaccines) that depend on high-quality, fresh-frozen tumor material.^8^ Despite these benefits, centers may face barriers in establishing robust snap freezing pathways.^7^ Another potential barrier to wider molecular neuropathology access is the time it takes to return molecular and genomic testing, with the Closing the Gap report identifying many potential bottlenecks within the WGS system in England that can delay the return of results.^7^ Because of the urgency of treatment for many patients with a brain tumor, clinicians need to make treatment and trial eligibility decisions rapidly, and can be discouraged from ordering tests such as WGS if results take many weeks or months to be returned.^7^ Finally, it is worth noting that because the NHS is devolved in the UK, each of the UK’s four nations (England, Northern ­Ireland, Scotland and Wales) have different commissioning arrangements for pathological testing, and only in England is WGS routinely funded for patients with neurological tumours.^7^

Given both the rapid advances in molecular neuropathology for neuro-oncology in recent years, and evidence that these advances are not being equitably adopted across the UK, it is vital to systematically explore the extent to which new approaches are being adopted and what barriers to patient access remain. We therefore analyzed longitudinal data collected from the Tessa Jowell Centre of Excellence program, to quantify the extent to which new technologies have been adopted. The Tessa Jowell Centre of Excellence program is an expert-led peer review process that collects data on the treatment, care and research delivered by centers in the UK who treat adult patients with a brain tumour.^9^ This review is a national benchmarking exercise open to all neuro-oncology centers in the UK, which collected data on many areas of the brain tumor pathway, including neuropathology. The existence of the National Health Service in the UK, although devolved to the four nations, makes such a review possible, as patients should (at least in theory) receive the same standard of treatment in every center commissioned to deliver neuro-oncological treatment and care. Data were collected in 2021 and again in 2024 to allow the identification of key trends across a three-year period.

The primary aim of this study was to assess and quantify the changing landscape of molecular neuropathology (including genomic testing, tumor snap freezing and analysis turnaround times) in the UK neuro-oncology centers from 2021 to 2024, using data from the Tessa Jowell Centre of Excellence review. Highlighting key areas of development, remaining challenges and areas of inequity in this period, three targeted recommendations to drive more equitable access to molecular and genomic testing are then proposed.

## Methods

### Data Collection as Part of the Adult Tessa Jowell Centre of Excellence Programme

28 of the 30 adult neuro-oncology centers in the UK applied in the first round of the Tessa Jowell Centre of Excellence for Adults Programme, with data collected between November 2020 and December 2021.^10^ A subsequent re-revaluation was conducted in 2024, covering 21 of these centers, with data collected between May and November 2024. Data were collected through an application form that covers 168 areas of the treatment, care and research activity relating to brain tumor patients.^10^ Questions included for molecular neuropathology are in supplementary file 1. While most data were based on local audits conducted by the applying team, certain data are local estimates, and this is noted throughout. Additional data collection and validation were by way of a 1.5-hour semi-structured interview with each applicant center, and feedback from patients with a brain tumor, collected through the Brain Tumor Charity’s ‘Improving Brain Tumor Care’ surveys.^11^

### Comparison of Neuropathology Data Collected in 2020/2021 and 2024

We compared submitted data from 21 centers collected in 2020/2021 (labeled “2021” in the rest of the paper) and 2024. The centers that applied in 2020/2021 but did not re-apply in 2024 were excluded for the purposes of this analysis and their data are not presented here; this decision was made in order to ensure representative averages could be generated for key statistics in 2021 and 2024. The 21 centers serve an estimated 84% of the UK population and include centers from every region and nation of the UK except Northern Ireland. The molecular neuropathology data collected from both application rounds included the annual number of samples submitted and turnaround time for the genetic analysis. Data on samples requested for methylation array, gene panel and WGS were collected in 2021 and 2024. Detailed data for fluorescence in situ hybridization (FISH), single nucleotide polymorphism array (SNP) and multiplex ligation-dependent probe amplification **(**MLPA) requests were collected in 2024, but not 2021. Detailed data on the time from surgical removal to snap freezing, number of samples snap frozen, and number of samples frozen to a volume of 1cm^3^ were available in 2024, but not 2021. Data were also collected in both 2021 and 2024 on the intraoperative (immediate analysis using histology), initial biopsy (rapid analysis for first patient discussions using immunohistochemistry) and final integrated diagnosis (including full molecular diagnosis) turnaround times (TATs). Additional data on the organization of genomic analysis, including auditing practice, organization within the GLH system, and whether cases were discussed within dedicated Genome Tumor Advisory Boards and multidisciplinary team meetings (MDT) were collected in 2024 but not 2021.

### Data Analysis

Completely anonymized quantitative data were extracted from questionnaires to allow comparisons between 2021 and 2024; due to the small sample size, formal statistical analyses were not conducted. Anonymized data are presented in descending value order and centers in each figure are numbered 1-21 (to clarify, this means that center numbers vary between figures, for example center 10 in Figure 2A is not the same as center 10 in Figure 2B). To compare the number of samples submitted for key molecular tests across centers, in some cases numbers were adjusted by glioma caseload per center (defined as number of new patients with a glioma seen by the center in a 12-month period; where used, this has been noted). Glioma caseload was selected because glioma molecular neuropathology testing is mandatory as part of the WHO CNS 2021 guidelines compared with skull base, pituitary tumors or metastatic disease.^1^

Any cases where complete data were not provided are noted on graphs and in figure legends (using an asterisk); missing data were not included in any calculated mean/medians or in average lines included on any figures (for this purpose, 2021 and 2024 were treated separately and a center was not excluded from the analysis of one year because a figure was not provided in the other year). Where a range was provided, the median was taken. All data were independently checked by two authors, and estimated data, or any outliers, were checked with the center in question as part of a data validation interview.

## Results

**Figure 1.**
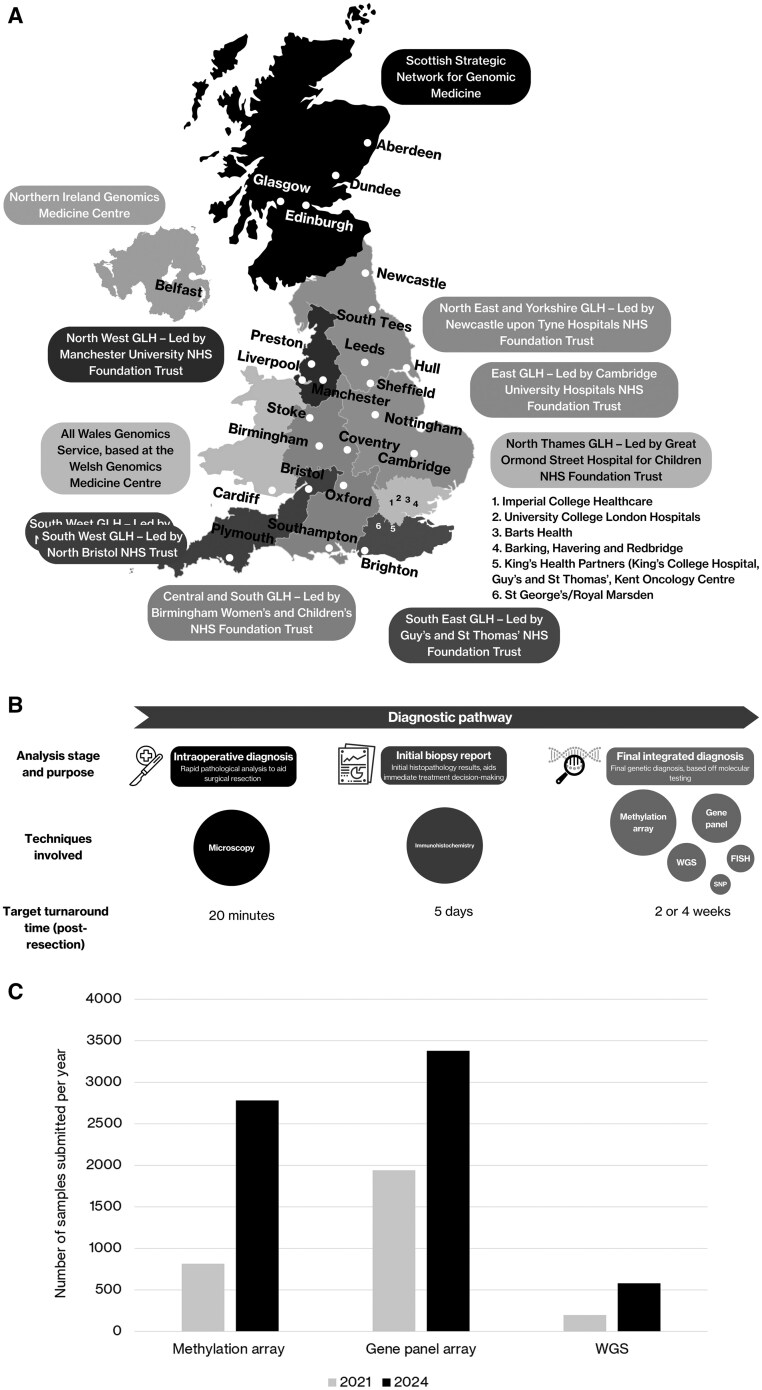
(A) Genomic testing capability in 2024 across the UK in 30 neuro-oncology centers and 10 regional networks, including English Genomic Laboratories Hubs (GLHs), All Wales Genomic Medicine Service based in the Welsh Genomics Medicine Centre, Scottish Strategic Network for Genomic Medicine and the Northern Ireland Genomics Medicine Centre. (B) Schema of neuropathological diagnosis and types of tests included in the standard neuro-oncology treatment pathway. Standards based on the Tessa Jowell Standards of Excellence. 14 days for in-house analysis from receipt in the genomic laboratory, 28 days for outsourced analysis (that is, analysis provided by a different institution, often in a different city, to where a patient’s treatment is being conducted). (C) Total number of samples submitted in the 21 centers in 2021 vs 2024 for methylation array, gene panel array and WGS testing of brain tumor tissue.

The UK’s 30 neuro-oncology centers are arranged into 10 networks with genomic testing capabilities, as shown in Figure 1A—7 English GLHs, the All Wales Genomic Medicine Service based in the Welsh Genomics Medicine Centre, the Scottish Strategic Network for Genomic Medicine and the Northern Ireland Genomics Medicine Centre. The data in this study were collected from 21 networks (including two centers who submitted a joint application), covering 84% of the UK population.

This study focused on the three key stages in the diagnostic pathway for molecular neuropathology (Figure 1B): intraoperative diagnosis, initial biopsy report and final integrated report incorporating molecular neuropathology. Each stage involves different testing methods, and relevant turnaround times are set out in the Tessa Jowell Standards of Excellence: ^10^ respectively, 20 minutes, 5 working days, and either 14 or 28 days respectively, with the timeframe for final integrated testing dependent on whether molecular diagnostic tests such as methylation array and gene panel are handled internally or externally. To note, the target TAT for WGS is 42 days.

### Rapid Growth in UK Genomic Testing between 2021-2024

The total number of glioma patients treated over the 21 ­centers increased 18% from 3159 in 2021 to 3722 in 2024. The total number of samples submitted for genomic testing (methylation arrays, gene panels, WGS, FISH, SNP, or MLPA) increased from 2946 to 6730 (128%) in the same period. The estimated molecular testing per glioma patient therefore increased from 0.9 to 1.8 from 2021 to 2024. Between 2021 and 2024, methylation array testing increased from 812 to 2779 (342%); gene panel testing increased from 1937 to 3377 (174%) and WGS testing increased from 197 to 574 (291%) (Figure 1C).

**Figure 2.**
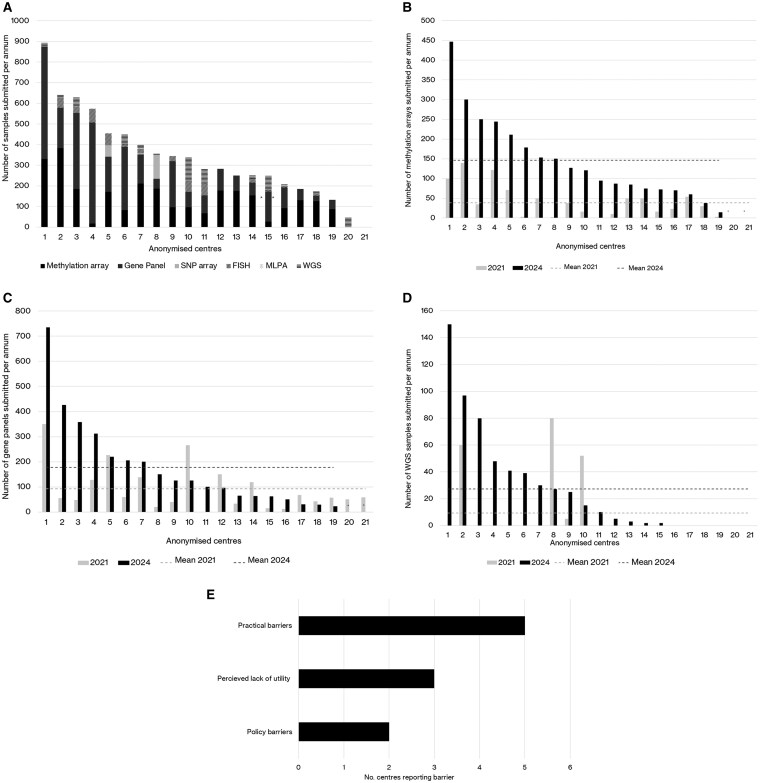
(A) Total number of samples submitted per each of the 21 centers for genomic testing of brain tumor samples in 2024, by genetic testing type in descending value order and adjusted for glioma caseload, in descending value order. Data adjusted for glioma caseload by the number of new glioma cases in the previous 12 months at that center, multiplied by the average number of new glioma cases across all 21 centers. * To note, center 20 data on WGS later in 2024 and did not report data for any other type of molecular testing; center 21 similarly only reported data for WGS (0 samples) and did not report data for other types of tests. (B) Number of brain tumor samples submitted for methylation array in the 21 centers in 2021 vs 2024 in descending order by 2024 value. *Centre did not provide data in 2024. (C) Number of samples submitted for gene panel arrays in the 21 centers in 2021 vs 2024 in descending order by 2024 value *Centre did not provide data in 2024. *Centre did not provide data in 2024. (D) Number of samples submitted in the 21 centers in for WGS in 2021 vs 2024 in descending order by 2024 value. (E) Reported barriers to providing WGS (*n*=8 centers reported barriers preventing any/some whole genome sequencing, some centers reported multiple barriers), identified in Question 1.3.7.

Data on the number of samples submitted for key tests were available in 2024 for 19 of 21 centers, as shown in Figure 2A adjusted for center glioma caseload; 2/21 centers only submitted WGS data. In 2024, methylation array was requested by *n* = 19/19 (100%) centers, gene panel by *n* = 19/19 (100%), WGS by *n* = 15/21 (71%), FISH by *n* = 13/19 (68%), SNP by 3/19 (16%) and MLPA by 0/19 (0%).

While there was an overall increase in samples submitted for key tests between 2021 and 2024 (gene panel, methylation array and WGS), the number of samples submitted across centers varied substantially (as demonstrated in Figure 2B-D). In the centers that submitted relevant data in both years, the number requesting methylation array and gene panel increased slightly—from 18/19 (95%) in 2021 to 19/19 (100%) in 2024 for both techniques, with an increase in the mean number of samples submitted for methylation array from 43 (median 35, range 0-139) in 2021 to 146 (median 121, range 15-447) in 2024, and in gene panel samples submitted from 92 (median 57, range 0-350) in 2021 to 178 (median 125, range 23-735) in 2024.

The number of centers requesting WGS increased more substantially, from 4/21 (19%) to 15/21(71%), with an increase in the mean samples submitted per center from 9 (median 0, range 0-80) in 2021 to 27 (median 5, range 0-150). For those centers unable yet to submit any (or more than a few) WGS samples, common barriers included practical barriers such as challenges in the testing pathway or consenting (5 centers), perceived lack of clinical utility (3 centers) or policy barriers outside of England (2 centers) (see Figure 2E).

### Brain Tumour Sampling with Snap Freezing in 2024, with Barriers

**Figure 3.**
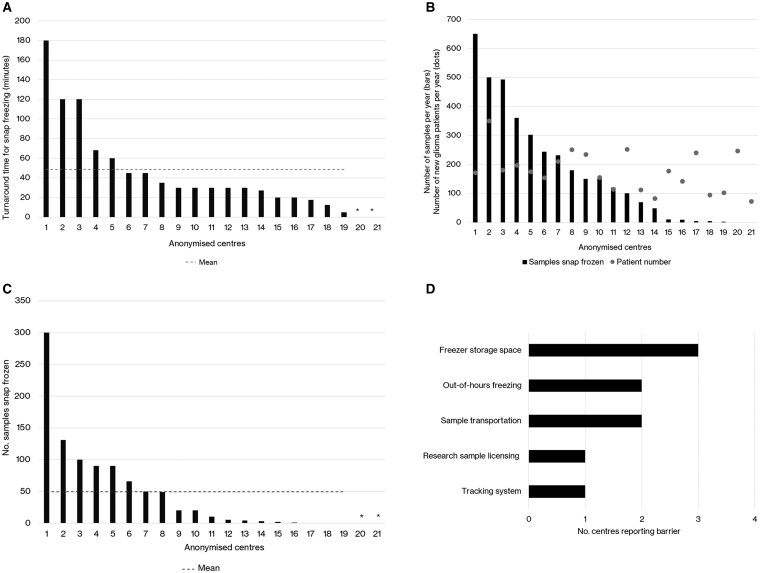
(A) Time to snap freezing (minutes) brain tumor tissue samples in 2024 in descending value order. *Centre did not provide data in 2024. (B) Number of brain tumor tissue samples snap frozen by center in descending value order across the 21 centers, and number of new patients with a glioma in 12 months, in 2024. (C) Number of brain tumor tissue samples snap frozen with a volume of 1 cm3 in 2024 in descending value order. *Centre did not provide data in 2024. (D) Reported barriers to snap freezing brain tumor tissue in 2024 (data collected from a free text response).

In 2024, 19/21 (90%) centers reported being able to snap freeze tumor tissue. The time interval between brain tumor tissue removal at neurosurgery and snap freezing ranged from 5 to 180 minutes (mean 49, median 30 minutes) in the 19/21 centers performing snap freezing (see Figure 3A). Centers reported a substantial range in the number of samples snap frozen (mean 173, median 120, range 0-650 samples; Figure 3B). Of the centers snap freezing any tissue, 16/19 (84%) collected the required 1 cm^3^ volume of tissue for at least some samples (see Figure 3C). Reported barriers to snap freezing material (see Figure 3D) included lack of freezer storage space (3 centers), difficulties with sample transportation to neuropathology laboratory for snap freezing (2 centers), lack of out-of-hours freezing (2 centers), tracking system challenges (1 center), and research sample licensing challenges (1 center).

### Turnaround Times for Intraoperative Diagnosis, Initial Biopsy Report and Final Integrated Diagnosis, Reported between 2021 and 2024 Compared with TJ Benchmark Standards

**Figure 4.**
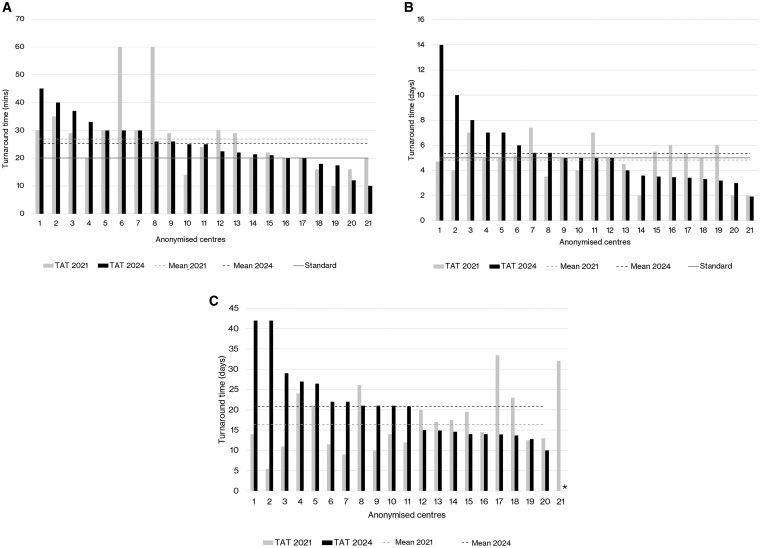
(A) Turnaround times in minutes for intraoperative diagnosis (from sample delivery at the pathology laboratory to reporting) in the 21 centers in 2021 vs 2024 in descending order by 2024 value. (B) Turnaround times in days for initial biopsy report (from collection of brain tumor sample to final report generation) in the 21 centers between 2021 vs 2024 in descending value order. (C)Turnaround times in days for final integrated diagnosis (from collection of brain tumor sample to final report generation)in the 21 centers in 2021 vs 2024 in descending value order. *Centre did not provide data in 2024.

TATs for the three key stages of the diagnostic pathway varied substantially across centers, as shown in Figure 4, in descending order 2021 vs 2024 across the 21 centers. The mean intraoperative TAT was 27 minutes (median 24, range 10-60) in 2021 compared with 25 minutes (median 25, range 10-45) in 2024 (Figure 4A). The 20-minute intraoperative TAT standard was met by 9/21 (43%) centers in 2021 vs 6/21 (29%) centers in 2024. The mean initial biopsy TAT remained relatively stable, at 4.8 (median 5, range 2-7) days in 2021 to 5.3 (median 5, range 2-14) days in 2024 (Figure 4B). In both 2021 and 2024 13/21 (62%) centers achieved the standard of an initial tumor biopsy TAT of 5 days or less. The mean TAT for final integrated diagnosis increased from 16 days (median 15 days, range 6-34) in 2021 to 21 days (median 21 days, range 10-42) in 2024 (Figure 4C). The 14-day TAT standard for final integrated diagnosis was met by 10/21 centers (48%) in 2021 vs 6/20 centers (30%) in 2024. In 2021 *n* = 2/21 centers (10%) exceeded a 28-day final integrated diagnosis TAT and in 2024 3/20 centers (15%) exceeded a 28-day final integrated diagnosis TAT.

**Figure 5.**
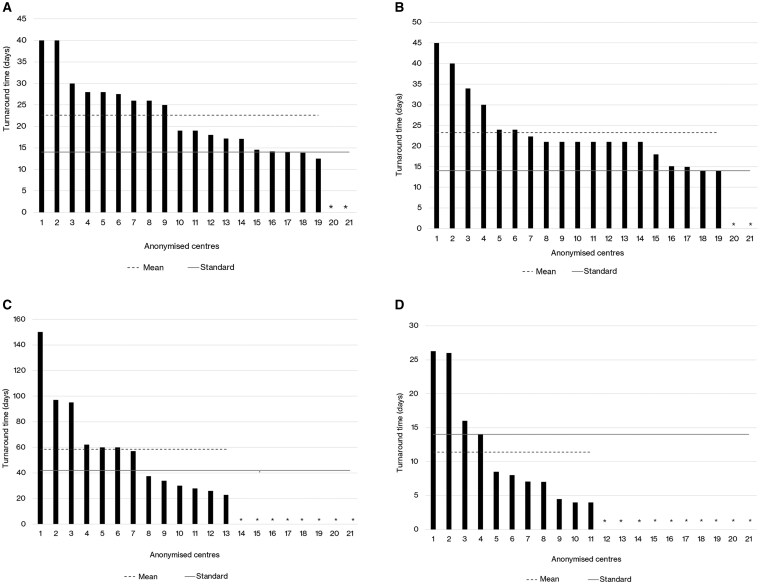
(A) Turnaround times for methylation array (from collection of brain tumor sample to final report generation) in days in descending value order in 2024 across the 21 centers. *Centre did not provide data or perform tests in 2024. (B) Turnaround times for gene panels (from collection of brain tumor sample to final report generation) in days in descending value order in 2024 across the 21 centers. *Centre did not provide data or perform tests in 2024. (C) Turnaround times for WGS (from collection of brain tumor sample to final report generation) in days by descending value order in 2024 across the 21 centers. *Centre did not provide data or perform tests in 2024. (D) Turnaround times for FISH (from collection of brain tumor sample to final report generation) in days by descending value order in 2024 across the 21 centers. *Centre did not provide data or perform tests in 2024.

Variations in TATs for final integrated diagnosis may be underpinned by variations in key tests including methylation arrays, gene panels, WGS and FISH; TATs for these tests varied substantially across centers, with relatively few centers meeting the Tessa Jowell Standards (Figure 5). The mean methylation array TAT was 23 days (median 19, range 13-40 days), with 3/19 centers (16%) meeting a 14-day TAT in 2024 (Figure 5A). The mean gene panel TAT was 23 days (median 21, range 14-45 days) with 2/19 (11%) meeting a 14-day TAT in 2024 (Figure 5B). The mean WGS TAT was 58 days (median 58, range 23-150 days), with 6/13 centers (46%) meeting the 42-day turnaround time for WGS in 2024 (Figure 5C). From the 11/19 centers with available data for FISH TAT in 2024 (58%), the mean FISH TAT was 11 days (median 8 days, range 4-26 days), with 8/11 (73%) meeting a 14-day TAT (Figure 5D).

### Embedding Molecular Neuropathology in UK Neuro-Oncology: Testing Infrastructure, Interpretation, and Audit Practices

While certain aspects of molecular and genomic testing in the UK are centralized, centers then discuss results locally; in 2024, 12/21 (57%) centers discussed genomic results in a dedicated Genetic tumor advisory board (GTAB) meeting, while 9/21 (43%) reported discussing results with a genomic section, neuropathology section or molecular pathology section within broader neuro-oncology MDT meetings (Supp. Figure 1). Among centers that held GTAB meetings, 8% (1 out of 12) met twice a week, 42% (5 out of 12) met weekly, 42% (5 out of 12) met every two weeks, and 8% (1 out of 12) met monthly.

Of note, in 2021, 6/21 (29%) centers reported carrying out molecular neuropathology audits vs 18/21 (86%) in 2024 (Supp. Figure 2). Of the 18 centers that conducted audits, 10/18 (56%) performed regular internal audits, while 8/18 (44%) relied on data from external organizations.

## Discussion

This is the largest population-level study comparing longitudinal anonymized molecular neuropathology data from 21 UK neuro-oncology centers serving an estimated 84% of the UK population collected in 2021–2024 as part of the Tessa Jowell Centre of Excellence for Adults Programme. These insights reveal key barriers to equitable molecular and genomic testing, which feed into three targeted recommendations.

### Despite Rapid Growth in Molecular Diagnostics for Brain Tumors, There Are Marked Geographic Disparities

The marked increase in genomic diagnostics for glioma patients between 2021 and 2024 (see Table 1) across 21 centers in the UK provides timely evidence of a system-wide shift to support precision oncology nationally, in line with UK policy ambitions.^12^^,^^13^ Over the three-year period of our study, the total yearly number of new patients with a glioma treated in the centers taking part in the Tessa Jowell Centre of Excellence program increased by 18% (from 3159 to 3722), while the number of genomic tests more than doubled (from 2946 to 6730), a 128% increase (see Figure 2A). Most strikingly, advanced molecular tests such as methylation arrays rose by 342%, gene panels by 174%, and WGS by 291%, with the proportion of centers offering WGS expanding from 19% to 71% (see Figure 2B-D). Our findings reinforce the strategic direction of the NHS Genomic Medicine Service (GMS), which aims to mainstream genomic testing for all patients with cancer as part of a national approach to personalized care.^12^ The uptake of gene panel and methylation profiling reflects clinical alignment with the 2021 WHO Classification of Tumors of the Central Nervous System, which now requires integrated histological and molecular diagnosis for accurate glioma classification.^1^

**Table 1.** Comparison of key molecular neuropathology statistics in 2021 vs 2024 for patients with a brain tumor in the 21 centers

Data (sample number or turnaround time, TAT)	2021 data	2024 data
Samples submitted for methylation array	Total 812, from 18/19 (95%) centers	Total 2779 from 19/19 (100%) centers
	Mean samples per center 43 (median 35, range 0-139)	Mean samples per center 146 (median 121, range 15-447)
Samples submitted for gene panel	Total 1937, from 19/19 (100%) centers	Total 3377, from 19/19 (100%) centers
	Mean samples per center 92 (median 57, range 0-350)	Mean samples per center 178 (median 125, range 23-735)
Samples submitted for WGS	Total 197 from *n* = 4/21 (19%) centers	Total 574 from 15/21(71%) centers
	Mean samples per center 9 (median 0, range 0-80)	Mean samples per center 27 (median 5, range 0-150)
Intraoperative TAT	27 minutes (median 24, range 10-60, 9/21 [43%] met 20 minutes standard)	25 minutes (median 25, range 10-45, 6/21 [29%] met 10 minutes standard)
Initial biopsy TAT	4.8 days (median 5, range 2-7, 13/21 [62%] met 5-day standard)	5.3 days (median 5, range 2-14, 13/21 [62%] met 5-day standard)
Final diagnosis TAT	16 days (median 15 days, range 6-34, *n* = 10/21 centers [48%] met 14-day target)	21 days (median 21 days, range 10-42, *n* = 6/20 centers [30%] met 14-day target)
Auditing—centers regularly auditing neuropathology data	6/21 (29%)	18/21 (86%)

However, the uneven distribution of testing across centers—particularly for WGS, with only 71% offering this test in 2024—highlights ongoing disparities in access to testing. Policy interventions may therefore be required to ensure equitable implementation of advanced diagnostics, potentially through regional investment strategies and NHS England’s leveling-up frameworks for genomic equity.^13^ Of particular note is the relative lack of access to genomic infrastructure in the devolved UK nations, which requires urgent high-level policy intervention.^7^ This inequity may prevent access to precision trials in certain parts of the UK, because these novel trials often require WGS as a criterion for entry.

### Disparities in Brain Tumor Sampling Are a Key Driver of Inequitable Access to Genomic Testing

The variability in the time interval between neurosurgical excision and snap freezing of brain tumor tissue, as depicted in Figure 3A, underscores inconsistencies across centers. While a median time of 30 minutes aligns with recommended practices for preserving molecular integrity in biospecimens, the upper range extending to 180 minutes may be associated with potential degradation of sensitive biomarkers. Prolonged post-excision intervals prior to cryopreservation may compromise tissue quality, thereby affecting downstream analyses, including transcriptomics and proteomics.^14^^,^^15^ The number of snap-frozen samples collected per center (Figure 3B) also varied markedly, suggesting geographic differences in infrastructure, staffing capacity, and institutional commitment to biobanking. While the mean sample number snap frozen per center in 2024 was 173, several centers reported low or zero snap freezing, impeding equitable access to high-quality biospecimens for national WGS genomic testing and national drug development initiatives.

Addressing insufficient snap frozen material submitted for diagnosis, genetics and emerging novel therapies has been highlighted previously and underscores the need for standardized pathways and the potential for targeted education campaigns for both patient and professional audiences.^16^ Importantly, the majority of centers that did snap freeze tissue (84%) were able to consistently meet the recommended tissue volume threshold of 1 cm³ (Figure 3C), which is considered optimal for multiple downstream analyses including short read WGS.^17^ Reported barriers to snap freezing provide critical insight into systemic challenges that may hinder sample collection. A lack of freezer storage space, cited by three centers, may reflect insufficient investment in biobanking infrastructure. Additionally, difficulties with transporting samples to the neuropathology laboratory and the absence of out-of-hours freezing support suggest that practical workflow constraints continue to limit optimal sample preservation (Figure 3D). These challenges are not unique to this study; previous national audits have similarly identified logistical and staffing barriers as major obstacles to high-quality tissue collection in real-world clinical settings.^18^ Less frequently reported but significant challenges include the lack of robust sample tracking systems and licensing issues. Inadequate tracking infrastructure can lead to sample misidentification or loss, undermining data integrity and reproducibility. Licensing concerns, while rare, reflect the complex regulatory landscape governing research use of human tissue.^9^ Addressing these operational and regulatory barriers will be essential to improve biospecimen workflows and ensure that samples are not only collected but are also usable under ethical and legal frameworks.^9^

### The Emerging Bottleneck in Diagnostic Turnaround Times Has Implications for Delivering Timely Clinical Care

Despite the increase in diagnostic capability, the overall TAT for final integrated molecular neuropathology diagnosis increased from a median of 15 days in 2021 to 21 days in 2024 (Figure 4C). The proportion of centers meeting a 14-day TAT benchmark decreased from 48% to just 30%. This may reflect system stress as test volumes increase and diagnostic pathways become more complex.^19^ Increase in TATs may negatively impact patients in terms of delays to the treatment pathway.^19^ National Institute for Health and Care Excellence (NICE) does not specify an exact time interval from surgery to radiotherapy/chemotherapy.^20^^,^^21^ However in clinical practice, particularly for high-grade gliomas, treatment is ideally started within 4-6 weeks post-surgery, depending on recovery and tumor histology. Moreover, delays beyond 6 weeks can negatively impact survival outcomes in aggressive tumors; ^20^^,^^21^ it is for these reasons that the Tessa Jowell Standards of Excellence stipulate that chemo/radiotherapy should be commenced within 4 and 6 weeks (respectively) from the decision to treat.^19^

Our TAT data highlight the need for targeted training in molecular pathology, genomic science and investment in novel technologies such as rapid long read technology to support sustainable service delivery.^5^ These data also raise important questions about workforce capacity in neuropathology, and genomic science.^22^ National audits, such as the Royal College of Pathologists’ workforce census, have previously highlighted shortages in consultant histopathologists and molecular scientists, particularly in regional centrs.^22^^,^^23^ Policy responses may need to address not only staffing levels but also training, including a consideration of automated interpretation strategies with Artificial Intelligence to maintain diagnostic performance alongside increasing demand.

### Embedding Molecular Neuropathology in UK Neuro-Oncology: The Evolving Landscape of Genomic Services Infrastructure, Interpretation, and Audit Practices

The landscape of genomic testing capabilities across the UK has evolved significantly in recent years, particularly within neuro-oncology centers. As depicted in Figure 6A, the UK's 30 neuro-oncology centers are organized into 10 networks with access to genomic testing: including seven English GLHs, the All Wales Genomic Medicine Service; the Scottish Strategic Network for Genomic Medicine, and the Northern Ireland Genomics Medicine Centre. This infrastructure reflects the national commitment to integrating precision medicine into routine clinical practice, particularly in neuro-oncology, where molecular diagnostics are becoming increasingly pivotal in guiding treatment decisions and prognostication.^24^^,^^25^

The integration of genomic data into multidisciplinary discussions has also seen notable changes. In 2024, 57% (12/21) of centers reported discussing genomic results in dedicated Genetic Tumor Advisory Board (GTAB) meetings, while 43% (9/21) integrated these discussions within broader neuro-oncology MDT meetings (see Supp. Figure 1). The frequency of GTAB meetings varied, with the majority (84%) meeting weekly or bi-weekly, reflecting an increasing emphasis on timely and specialized review of complex molecular data. This shift towards dedicated genomic-focused discussions aligns with emerging best practices, which advocate for specialized molecular tumor boards to interpret and contextualize genomic findings.^26^ The disparities in how genomic data are discussed—either in dedicated GTABs or within broader MDTs—may reflect differences in institutional resources, expertise, and patient volumes. While dedicated GTABs provide a focused platform for in-depth genomic analysis, integrating genomic discussions into MDTs can promote a more holistic approach, ensuring that molecular data is considered alongside clinical and radiological findings.

Interestingly, the increase in molecular neuropathology TAT audits from 29% in 2021 to 86% in 2024 (see Supp. Figure 2) underscores a growing commitment to quality assurance and continuous improvement in genomic diagnostics among those centers engaging in the Tessa Jowell Centre of Excellence program. Of the centers conducting audits, 56% performed regular internal reviews, while 44% leveraged external data sources. Regular auditing is crucial for maintaining the accuracy and reliability of genomic testing, especially as the field rapidly evolves and new biomarkers and technologies emerge.^27^ These audits not only ensure compliance with national guidelines but also facilitate benchmarking and sharing of best practices across the network.

### Underlying Causes, Potential Solutions and Strategic Recommendations

This study has identified significant regional disparities in access to molecular diagnostics in the UK. As elucidated in previous work,^7^ there are three core challenges underpinning these disparities, each of which require a different policy response:

1. Resourcing and logistics: many centers face challenges in delivering a timely service due to the shortage of key neuropathological staff noted in recent national workforce audits^22^^,^^23^ as well as logistical issues within the GLH system noted in the Closing the Gap report; ^7^
2. Perceived lack of utility: logistical and resourcing challenges have led to some clinicians expressing skepticism about the utility of genomic testing,^7^ given the time required and potential for delays to render results not clinically relevant;
3. Policy choices: a clear gap outside England is the lack of centralized commissioning and funding of genomic testing, with patients treated in Northern Ireland, Scotland and Wales lacking ready access to testing that is available in England.^7^

From a national perspective, there are several steps that can be taken to overcome these challenges, to ensure that all patients in the UK can access the same high standard of treatment available on the NHS:

1. Ensure sustainable service delivery: To address both the perceived lack of utility and resourcing challenges, to ensure a sustainable service, investment is needed in targeted training in molecular pathology and genomic science, combined with adoption of novel rapid technologies.
2. Address equity of access: Geographic disparities in brain tumor sampling and test availability suggest the need for standardized pathways and targeted interventions to ensure equitable distribution of genomic testing in the whole of the UK, overcoming both resourcing challenges and the policy challenge.
3. Monitoring and accountability: Participation in the Tessa Jowell Centre of Excellence program, with regular internal audit of TAT demonstrates a commitment to improve and achieve ideal clinical standards, and should be continued, to support ongoing national service improvement

In conclusion, the evidence presented here supports UK policy ambitions to embed precision diagnostics across brain cancer care. It also underscores the need for coordinated implementation strategies that address both molecular neuropathology capacity, adoption of novel rapid technologies, and equity, to fully realize the clinical therapeutic benefit for patients with a brain tumor.

## Supplementary Material

npaf099_Supplementary_Data

## Data Availability

Data will be made available upon reasonable request to the authors.
