# Supplementary material for: Transforming molecular neuropathology for adult brain tumor patients in the UK: Insights on implementation, adoption, and patient access (2021-2024)
Source: Neurooncol Pract. 2025 Sep 25;13(3):465–77. doi: 10.1093/nop/npaf099 (PMC13161907; doi:10.1093/nop/npaf099)
Supplement: npaf099_Supplementary_Data [file npaf099_supplementary_data.zip › REVISED Supplementary File.docx]

**Supplementary Figure 1:** Neuropathology section of the Tessa Jowell Centre of Excellence for Adults application form, distributed to all UK neuro-oncology centres between February and May 2024.

- 1. Pathology *(To be completed by a neuropathologist)*

1.3.1. Outline if you meet the following requirements:

| Requirement | **If “yes”, provide date of certification. If “no”, elaborate:** |
| --- | --- |
| Are your specialist neuropathology laboratory facilities and/or genomics laboratory hubs United Kingdom Accreditation Service (UKAS) accredited to International Organisation for Standardisation (ISO) 15189? *Please insert date of accreditation and number.* |  |
| Are all neuropathology reports created by specialist neuropathologists taking part in the External Quality Assessment (EQA) scheme? *Please insert date of certification.* |  |

- - 1. Please describe the neuropathology team set up at your centre. Include number of staff and estimated job plan allocated to neuro-oncology cases (in FTE). [Exploratory] (150)

|  |
| --- |

- - - 1. To understand neuropathology turnaround times, please complete the following the table:

| **Type of analysis** | **Average turnaround time** (minutes/days) | **Analysed in house? (Y/N)**  If no, indicate where samples are analysed |
| --- | --- | --- |
| Intraoperative diagnosis |  |  |
| Initial interim tumour biopsy report, prior to integrated analysis with genetics |  |  |
| Final integrated diagnosis *(including genetics if relevant)* |  |  |

- - - 1. Please share the date of your most recent audits, or relevant data source to evidence the above turnaround times. (80)

|  |
| --- |

- - 1. Briefly describe the processing pathway (“chain of custody”) for neuropathology samples from surgery to laboratory analysis. Are samples tracked from surgery to laboratory and is the time of receipt of samples recorded? (100)

|  |
| --- |

**Snap frozen tissue**

- - 1. Describe the process by which samples are snap frozen in your centre. Explain any barriers (e.g., freezer capacity). (80)

|  |
| --- |

- - 1. Please provide the following metrics within your snap freezing pathway:

| **Metric** | **Estimate** |
| --- | --- |
| Number of tumour samples snap frozen in 2023 (or specified 1-year time frame) |  |
| Number of cases where viable tissue is frozen at a volume of 1cm x 1cm x 1cm |  |
| Time it takes for a sample to be snap frozen following neurosurgical removal |  |

**Genomics**

- - 1. Please indicate which Genomic Laboratory Hub (GLH) or equivalent Genomics Medicine Centre for Scotland, Wales and Northern Ireland you are associated with (if any). Please describe how you communicate and work with your local GLH (or regional equivalent) and any challenges: (80)

|  |
| --- |

| **Genomic tests** | **Number of samples submitted/year** | **Average turnaround time** (days) | **Analysed in house? (Y/N)**  If no, indicate where samples are analysed |
| --- | --- | --- | --- |
| Methylation array |  |  |  |
| Gene panel + relevant fusions |  |  |  |
| SNP Array |  |  |  |
| FISH |  |  |  |
| MLPA |  |  |  |
| Whole genome sequencing |  |  |  |

1.3.8.1. Please detail below how genomic testing is organised in your centre.

- - - 1. Please share the date of your most recent audits, or relevant data source to evidence the above turnaround times.: (80)

|  |
| --- |

- - 1. Are genomic results discussed in a molecular tumour board (MTB) or genomic tumour advisory board (GTAB)? If yes, please describe meeting frequency and whether they are run at your centre or elsewhere. Please note any challenges experienced. [Exploratory] (150)

|  |
| --- |

**Supplementary Figure 2:** Pie chart showing number of centres (and percentage) with discussion of molecular neuropathology results in dedicated Genomic Tumour Advisory Boards versus General Multidisciplinary Team Meeting (MDT) in 2024; data collected from a free-text question.

**Supplementary Figure 3:** Bar chart comparing auditing of neuropathology service (e.g. turnaround times and tissue sample collection/snap freezing) across the 21 centres in 2021 vs 2024.
